# Vulvar Lichen Sclerosus from Pathophysiology to Therapeutic Approaches: Evidence and Prospects

**DOI:** 10.3390/biomedicines9080950

**Published:** 2021-08-03

**Authors:** Monica Corazza, Natale Schettini, Pierantonia Zedde, Alessandro Borghi

**Affiliations:** Section of Dermatology and Infectious Diseases, Department of Medical Sciences, University of Ferrara, 44121 Ferrara, Italy; czm@unife.it (M.C.); schntl@unife.it (N.S.); zddpnt@unife.it (P.Z.)

**Keywords:** vulvar lichen sclerosus, etiopathogenesis, pathway, immunity, fibroblasts, collagen metabolism, therapy, corticosteroid

## Abstract

Vulvar lichen sclerosus (VLS) is a chronic, distressing, inflammatory disease with an enormous impact on quality of life. Treatment goals are relieving symptoms, reversing signs and preventing anatomical changes. Despite the availability of numerous therapeutic options, treatment outcome may not be entirely satisfactory and a definitive cure does not exist. This may be due to the fact that the exact VLS etiopathogenesis remains unknown. The objectives of this paper were to review the most up-to-date knowledge on VLS etiopathogenesis and to consider the available therapies through the lens of a plausible pathogenetic model. An electronic search on both VLS etiopathogenesis and its treatment was performed using the National Library of Medicine PubMed database. Based on current knowledge, it is conceivable that various, heterogeneous environmental factors acting on a genetic background trigger an autoimmune, Th-1 response, which leads to a chronic inflammatory state. This, in turn, can determine both tissue and micro-vascular injury and activation of signaling pathways involved in fibroblast and collagen metabolism. This pathogenetic sequence may explain the effectiveness of anti-inflammatory treatments, mostly topical corticosteroids, in improving VLS clinical-pathological changes. Further deepening of the disease pathways will presumably allow key mediators to become new therapeutic targets and optimize the available treatments.

## 1. Introduction

Lichen sclerosus is a chronic inflammatory progressive skin disease. It mostly affects the anogenital areas [1,2]. The disease occurs at all ages and in both sexes, but is more common in females than in males. The exact prevalence of vulvar LS (VLS) is unknown but is estimated to range between 0.1% and 3% in the pre-pubertal and postmenopausal periods, respectively [2,3]. Regardless of its exact incidence, VLS is among one of the most common referrals for vulvar distress and/or structural changes [4].

VLS primary lesions are flat, ivory or porcelain white spots, which may coalesce into pale, crinkly, thin patches and plaques. Erythema, ecchymosis and itching-related excoriations may occur and, occasionally, hyperkeratosis is prominent. 

The sites most characteristically involved by VLS are the inter-labial sulci, labia minora and labia majora, clitoris and clitoral hood. The entire vulva may be involved, with possible extension to perineum and perianus, giving rise to the characteristic ‘figure-of-eight’ shape. The genitocrural folds, buttocks and thighs may be affected as well. The vagina is usually spared. The main differential diagnoses are lichen planus, lichen simplex chronicus, vitiligo, immunobullous disorders such as mucous membrane pemphigoid and vulvar intraepithelial neoplasia.

Post-inflammatory progressive scarring may cause irreversible subversion of anogenital architecture, including fusion or loss of the labia minora, narrowing of the vaginal introitus and burying of the clitoris.

There is also an increased risk of genital cancer. In fact, women affected with VLS have a lifetime risk of developing a squamous cell carcinoma estimated to be 2–5%, while up to 65% of vulvar carcinomas arise in a background of VLS [5,6,7,8].

The diagnosis of VLS is clinical, but a biopsy may be necessary when the clinical picture is not diagnostic. Typical histological features consist of orthohyperkeratosis, loss of rete ridges, epidermal atrophy, basal cell degeneration, dermal hyalinization with a homogenized band of dense fibrosis at papillary dermis and a band-like lymphocytic infiltrate [9]. In the early phases, VLS poses a diagnostic challenge since it presents as an interface dermatitis without the classic papillary dermal homogenization [10]. Dermoscopic examination may support a non-invasive diagnosis [11] as well as enhance differential diagnosis with respect to other inflammatory and neoplastic vulvar disorders [12]. A prompt diagnosis seems to have favourable consequences on the course and prognosis of VLS, since previous experiences have found that early intervention with topical corticosteroids may revert skin changes and prevent both scarring and tumour evolution [13,14]. In spite of this, delayed diagnosis in VLS is common [15].

Most patients with VLS complain of distressing symptoms which include itching, burning, stinging and pain, the latter especially elicited by sexual intercourse [1,2,13,15,16,17,18]. Chronic inflammation, anatomic changes and presence of erosions and fissures are the main causal factors of sexual pain and apareunia, which are frequently complained of by patients [15,19,20].

Growing evidence has shown that VLS has a great impact on the well-being and quality of life of affected subjects. Its annoying symptoms, chronic course, sexual dysfunction, disfiguring anatomical changes, partial and temporary response to treatment and risk of evolution towards cancer, are the main determinants of VLS-related burden [21,22,23,24,25,26].

In spite of these huge detrimental effects on health, a definitive cure for VLS does not exist, since its exact etiopathogenesis remains unknown [1].

The present paper aims to analyse the most up-to-date knowledge on the etiopathogenesis and treatments of VLS through a full extensive review of the literature. The etiopathogenetic framework of this disease may be the perspective from which to consider the current therapeutic options and, ideally, address new ones.

All the studies evaluating either VLS etiopathogenesis or VLS treatment published in the English literature were analysed in April 2021, with no date limitations. An electronic search was performed using the National Library of Medicine PubMed database. Papers not written in English were excluded. All studies identified as relevant, including research articles, controlled studies, case series, guidelines, reviews and single case reports that raised important issues were included in the present review.

## 2. Update on VLS Etiopathogenesis

To date, the etiology and pathogenesis of VLS are not well defined. The available evidence indicates the role of two main pathomechanisms, which act on a susceptible genetic background. Suspected triggering factors complete this pathogenetic scenario.

More in detail, immunological dysreactivity and chronic inflammation represent one of the two aforesaid key pathomechanisms. On the other hand, promoting fibroblast growth and activity—as well as abnormal collagen synthesis, which leads to a progressive formation of hyalinised and sclerotic dermal tissue—is the other crucial event in VLS pathogenesis [27,28].

It is conceivable that in VLS, as in other dermatological chronic inflammatory disorders, immune dysreactivity and pathological changes are closely associated. However, the link between these two events and their exact sequence, as well as the role of potential triggers, have not yet been clarified. All the protagonists of this intricate scenario are analysed below.

### 2.1. Predisposing Background and Genetics

A family history of LS has been shown in several studies [29,30,31,32,33]. The rate of first-degree relatives affected with LS ranges between 5.4% and 12% of the patients. Case reports of LS in monozygotic and dizygotic twins support a genetic component as well [34,35].

A significant positive association with genes regulating HLA class II antigens further strengthens the view that there is a genetic susceptibility to VLS [36]. In particular, HLA-DQ7, -DQ8, -DQ9 and -DR12, mostly haplotype DRB1*12/DQB1*0301/04/09/010, are shown to appear more frequently in LS patients than in controls. Associations between LS and other both class I and class II antigens, namely HLA -B*08–B*18, -B*15, -B*57, -CW*03, -CW*07, -CW*18, -DRB1*04, -DRB4*, -DRB1*07, -DRB1*12 have been documented [30,36,37,38]. On the other hand, some HLA antigens appear involved in protection from LS [36].

In addition to predisposing HLA haplotypes, both changes in the genomic sequence and epigenetics, i.e., functionally relevant changes in gene expression or cellular phenotype, have been described in VLS. A recent study profiled the genomes of VLS patients in comparison with an unaffected relative through the whole-exome sequencing technique [39]. It found recurrent germ-line variants in genes encoding for proteins playing immunomodulatory and/or tumour suppressor activities, such as CD177, CD200, ANKRD18A and LATS2, which can be putative contributors to VLS pathogenesis. A number of further DNA sequence and epigenetic alterations of some genes, mainly oncogenes, have been described in VLS as well as in vulvar carcinomas associated with LS. These include, among others, CDKN2a, TP53, KRAS, DAPK1 and RARβ [27,28,40,41,42,43,44,45,46,47,48,49,50]. It has been hypothesized that aberrant immune response, and collagen synthesis as well, may have a genetic or epigenetic background [28,51,52]. As in any multifactorial disease, in VLS a genetic or epigenetic predisposition reflects the cumulative effect of variations at multiple loci sequence and/or expression, which leads to susceptibility to disease. Susceptibility requires interaction with exogenous factors to result in disease.

### 2.2. Immune Dysregulation and Inflammatory Response

Several lines of evidence suggest a pivotal role of autoimmunity in VLS pathogenesis. A higher percentage of VLS patients, when compared with controls, have one or more autoimmune-related disease or a family history of autoimmune disease or autoimmune antibodies [53,54,55,56,57,58,59,60]. Thyroiditis, alopecia areata, vitiligo and pernicious anaemia are the most common autoimmune diseases diagnosed in patients with VLS. Autoimmune bowel disease, localized scleroderma/morphea, rheumatoid arthritis, systemic lupus erythematosus and multiple sclerosis are less frequent associations.

Immunological dysreactivity and autoimmune response appear relevant pathophysiological mechanisms. More in detail, an abnormal activation of a Th1 autoimmune response has been found in affected tissue [61,62]. Th1-type pro-inflammatory cytokines and immune-mediators, such as IL-1, IL-7, IL-15, IFN-γ, TNF-α, IL-2 receptor (CD25), caspase 1, ICAM-1 and its ligand CD11a, are recognized to be up-regulated in LS. This supports the likelihood that a Th1 reactivity may promote the development of VLS. An increased tissue expression of CXCR3 and CCR5, chemokine receptors known to characterize a Th1 response, is in line with this hypothesis [61,62].

On the other hand, a concurrent impairment in Treg activity may be assumed in VLS tissues. Treg cells are recognized to have a critical role in the maintenance of immune tolerance. Therefore, a reduced Treg function leads to both impaired immune tolerance toward self-antigens and autoimmunity. An overexpression of microRNA-155 (miR-155) in LS lesions compared to controls has been supposed to induce a suppression of Treg cell activity [61,63]. The low expression of Foxp3, which is a marker and a function regulator of Tregs, in the affected sites is a further possible determinant of the impaired immunosuppressive function of this lymphocytic subtype in VLS [52]. A decrease of circulating CD127, a specific phenotypic marker of CD4+ CD25+ Treg cells, in the blood of VLS subjects, compared with healthy controls also suggests a systemic dysfunction of Tregs. Data inconsistent with these are also available, highlighting the complexity of the immune mechanisms and the need of further investigation [64].

In keeping with a likely reduced Treg cell activity, low IL-10 levels have also been found in LS [28]. Taken together, these determinants and markers of abnormal immune control create a microenvironment conducive to autoimmunity [28,61,63].

This immune imbalance perpetuates an up-regulation of pro-inflammatory cytokines and, in turn, leads to a cell-mediated attack against self-antigens. In fact, in lesional tissue a high number of activated T cells that express perforin, Tia1, and granzyme B have been found [65,66]. Thus, a T cell-mediated cytotoxicity seems to play a major role in the pathogenesis of LS and is sustained by the chronic release of pro-inflammatory cytokines, mostly IFNγ [62]. The local expression of the interferon-inducible protein IP10/CXCL10 seems to act in the recruitment of CXCR3+ cells into the skin [61,66]

Chronic inflammation also induces the production of reactive oxygen species (ROS), which are deemed responsible for contributing to tissue damage [67,68]. An increase of lipid peroxidation products, particularly within the basal layers of the epidermis, and oxidative DNA and protein damage are direct consequences of the oxidative stress contextual to LS [68]. Oxidative processes are putative contributors to autoimmunity, tumorigenesis, tissue sclerosis and scarring. These latter lead to constriction and damage of dermal vasculature which, in turn, further maintain and increase local oxidative stress. ROS, and chronic tissue damage more in general, also seem to create new skin epitopes. These enhance the development of autoantibodies, especially in subjects with an autoimmune diathesis. Autoantibodies to extracellular matrix protein 1 (ECM1) and to the basement membrane zone BP180 and BP230 have been found in LS patients [69,70,71,72,73]. ECM1 autoantibodies potentially affect the functional binding of ECM1 to both collagen IV and other basement proteins at the dermal-epidermis junction and, to a lesser extent, matrix metallopeptidase 9 (MMP-9), resulting in the focal basement membrane disruption observed in LS [74]. Moreover, ECM1 has a role in the structural organization of the dermis and controls keratinocyte differentiation and angiogenesis. Loss-of-function mutation studies in ECM1 gene in lipoid proteinosis, an autosomal recessive genodermatosis that shows comparable clinical and histopathological features to those of LS, implicates ECM1 as a strong putative autoantigen in LS autoimmunity [75]. However, at the current state of knowledge, it seems more plausible that the formation of autoantibodies and humoral autoimmunity are an epiphenomenon, i.e., a consequence of the formation of novel, previously sequestered tissue epitopes, rather than primary pathogenetic mechanisms [71]. This phenomenon, called epitope spreading, consists in an immune response induced by previously hidden antigens, which become exposed, and recognized by the immune system, after tissue destruction via an autoimmune or inflammatory process.

### 2.3. Abnormal Collagen Metabolism

Promoting fibroblast growth and activity, as well as abnormal collagen synthesis, is another crucial event in VLS pathogenesis and progressive formation of hyalinised and sclerotic dermal tissue [28]. Several pathways seem to be involved in disrupting fibroblast and collagen homeostasis. An miR-155 overexpression has been found to decrease expression and activity of two tumour suppressor genes, namely FOXO3 and CDKN1B51. Decrease in FOXO3 and CDKN1B expression seems to promote fibroblast proliferation. Thus, miR-155 may contribute to the pathogenesis and development of VLS both inducing autoimmunity and enhancing fibroblast proliferation.

TGF-β, which stimulates extracellular matrix deposition and is a known mediator of fibrosis in scleroderma, was found overexpressed in a few studies [76,77,78], but strong evidence is lacking on its involvement in VLS.

Growth differentiation factor-15 (GDF-15), which is a distant member of the TGF-β superfamily, promotes fibroblast activation and has a crucial function in the pathogenesis of fibrotic diseases [79]. In accordance with this, the serum levels of GDF-15 are increased in patients with systemic sclerosis, above all in those with diffuse cutaneous forms [80]. A significant increase in GDF-15 levels has recently been observed in VLS fibroblast in comparison with healthy fibroblasts [81]. This finding further highlights the critical role of fibroblasts in VLS physiopathology via multiple pathways, including GDF15 protein secretion

An involvement of matrix metalloproteinases has also been supposed: mostly MMP-9 and MMP-2, and their inhibitors, i.e., tissue inhibitors of metalloproteinases (TIMPs), whose interaction is essential for maintaining the balance between extracellular matrix (ECM) synthesis and degradation [82]. In particular, an increase in the immunodistribution of MMP-2 and -9 and TIMP-1 and -2 in VLS compared to normal vulvar skin has been observed, suggesting their role in collagen remodelling found in this disease. Modulation in decorin expression within VLS dermis may reflect the abnormal MMP activity as well as contribute to pathological matricial remodelling [83].

Further potential actors of these histopathological changes are collagen V (COLV) and galectin-7 which both appear increased in VLS [84,85]. Increased COLV expression, and contextual ECM1 decrease, may contribute to both the disappearance of elastic fibres in the upper homogenized dermis and the abnormal collagen metabolism. On the other hand, the increased expression of galectin-7 in patients with VLS compared with healthy tissue may influence the viability of fibroblasts. In fact, the synthesis of collagen I and collagen III, which are the primary collagens generated by dermal fibroblasts, is stimulated by galectin-7 in a dose dependent manner. Thus, the hyalinization mediated by fibroblasts may be significantly enhanced by galectin-7.

### 2.4. Triggering Factors

Several agents have been investigated as possible causal or triggering agents of LS. Convincing evidence is lacking but it cannot be excluded that exogenous agents can trigger immune activation and inflammation, initially even in a non-specific way. Then, especially in the case of prolonged action of the potential trigger and in predisposed subjects, the inflammatory process may assume the typical phenotype underlying LS. Therefore, on the basis of current knowledge, if an actual etiological role *stricto sensu* cannot be attributed to any specific agent, the effect of exogenous stimuli on activation and perpetuation of an immune response and inflammatory state appears plausible. Several heterogeneous factors could induce autoimmune responses via different mechanisms. This may explain the controversial and not univocal results arising from the research conducted in this regard.

Infectious agents, especially Borrelia burgdorferi [86,87], human papilloma virus (HPV) [88], hepatitis C virus [89] and Epstein–Barr virus [90], have been researched in either the skin or blood from LS patients with conflicting and in no case conclusive results. The possible consequences of SARS-CoV-2 virus infection on the clinical course of VLS are not known. In fact we have not had any known cases of VLS patients with intercurrent infection with this virus, nor have there been any cases reported in the literature.

A dysbiosis has been observed in the skin and gut microbiotas of LS subjects [91]. In particular, the bacterial genera Prevotella spp., Peptostrepococcus spp., Porphyromonas spp. and Parvimonas spp have been found to be more abundant on several skin districts of girls with LS compared to healthy individuals. In the gut samples, significantly higher relative abundance of Dialister spp., Clostridiales spp., Paraprevotella spp., Escherichia coli, Bifidobacterium adolescentis and Akkermansia muciniphila was observed in patients than in controls. Alterations in both cutaneous and gut microbiotas may support inflammatory processes in LS, as in other inflammatory skin conditions, by modulating systemic immunity [92,93,94].

Trauma and chronic irritation have been postulated to lead to LS development. The Koebner phenomenon, also termed isomorphic response, which describes the occurrence of clinically and histologically disease specific lesions on normal appearing skin after trauma, is described in LS [2]. Mechanical factors like friction due to tight clothing, occlusion, sexual intercourse, tissue damage during childbirth, genital jewellery and piercing, surgery, radiotherapy and scars, in general, are thought to trigger and maintain genital LS [31,95,96,97,98,99,100,101].

Chronic irritation from exposure to urine as well as, in males, from the occlusion and moisture determined by the prepuce, are further conditions permissive for the development of genital LS [102]. 

In addition to exogenous determinants, also possible endogenous factors have been proposed as conditioning the development of LS. For instance, a hormonal influence has been supposed. In particular, a defect of androgen action, due to low circulating levels or decreased specific receptors or, again, iatrogenic suppression, has been supposed to enhance the development of the disease [103,104,105].

Overweight, obesity and blood hypertension were more frequent among a large cohort of LS patients than among the general population, suggesting dysmetabolic conditions to be possible triggers in predisposed individuals [29]. 

### 2.5. A Possible Pathogenetic Model

**Figure A1:**
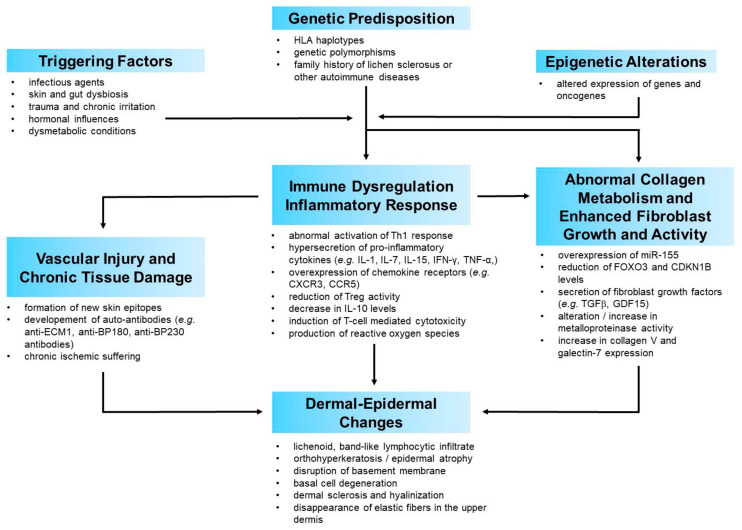
Proposed pathogenetic model of vulvar lichen sclerosus.

The current evidence on physiopathology of VLS points towards a complex, multifactorial process. Once the possible factors involved in the pathogenesis of VLS have been focused, it is possible to try to define a credible scenario in which the interaction of the aforementioned factors leads to the development of this disease. It is conceivable that environmental factors acting on a genetic background trigger autoimmune processes, with subsequent inflammation which, in turn, leads to tissue and microvascular injury as well as to activation of signalling pathways involved in fibroblast and collagen metabolism. Dysregulation of pro- and anti-fibrotic mechanisms determines the degree of dermal fibrosis and all the distinctive histopathological features of LS, which represent the culmination of this process (Figure A1).

Exogenous triggers may act as generic damage-associated molecular patterns (DAMPs), which ignite the immune response. This activates an inflammatory cascade with raised levels of pro-inflammatory cytokines predominantly belonging to a Th1 type of inflammation. A recent research quantified the cytokine/chemokine levels in independent keratinocyte and fibroblast cultures derived from VLS samples and healthy controls [81]. The cytokines/chemokines which resulted most dysregulated in VLS keratinocytes and/or fibroblasts were DKK1, a soluble antagonist of β-catenin-dependent Wnt signaling, growth differentiation factor-15 (GDF-15), a member of the TGF-β superfamily, insulin-like growth factor binding protein-2 (IGFBP-2) and chitinase-like protein Chi3L1. The most noteworthy aspect of these findings is that all these mediators are implied in both inflammatory processes and collagen/fibroblast metabolism. They share the common characteristic of being a sort of “bridge” between inflammation and collagen metabolism. This not only confirms the implication of both these pathways but also their close crosstalk in the pathophysiology of VLS. The involvement of certain mediators which favour the interplay between inflammation and fibroblast biology, or which are themselves junction points between these two systems, could be crucial in switching the primary inflammatory process into the gradual development of the histological and clinical changes characteristic of VLS. This seems to be confirmed by the histopathological close approximation of immune cell infiltrates with sclerosis in VLS tissue, further supporting the interplay between immune dysregulation and increased extracellular matrix components.

In this perspective, from a chronological point of view, inflammation may be the first pathogenetic event in VLS, followed by an alteration of the collagen metabolism, which is the clinical-histological epiphenomenon of this process. In accordance with this view, histologically early LS is typified by a lichenoid, lymphocyte-predominant band of inflammation, basal vacuolization and focal cytotoxic damage to basilar keratinocytes. Progressively over time, the inflammatory infiltrate is displaced by dermal oedema and homogenization of papillary dermal collagen fibres [106,107].

This sequence would explain the efficacy of anti-inflammatory treatments, especially topical corticosteroids or topical calcineurin inhibitors, in significantly improving dermal collagen changes [108]. In some ways, this resembles the histological and clinical improvement obtained in other different immune-mediated inflammatory skin diseases following the administration of molecules capable of blocking the immune mechanism. Psoriasis, pemphigus vulgaris, lichen planus, hidradenitis suppurativa or drug-induced cutaneous adverse reactions are just some examples of skin diseases in which clinical-histological healing is obtained by antagonizing the immune and inflammatory cascades [109,110,111,112,113]. In all these diseases, as well as in many other immune-mediated skin diseases of unknown aetiology, nonspecific triggering factors activate the immune cascade. For instance, Koebner phenomenon is a shared trigger for the development of several morphologically different skin diseases such as psoriasis, lichen planus, vitiligo, LS and others [114]. In a similar way, other nonspecific factors may trigger the occurrence or relapse, or worsening of heterogeneous skin conditions. These include infectious agents, drugs, emotional distress or dysmetabolic syndrome. What differs from one disease to another is the triggered and activated immune pathway. The immune pathway elicited in each disease appears to be driven by a genetically determined background [115,116,117,118,119,120,121,122]. The different immune mediators involved account, in turn, for the pathogenetic, histological and clinical differences among these diseases.

Thus, in our view, the main pathomechanisms of VLS, despite the differences, may be similar to those of other chronic inflammatory multifactorial diseases. Genetic or epigenetic background determine the activation of a peculiar immune cascade, and the release of the relative mediators, which is elicited by unspecific factors that probably simply act as triggers. The type of activated immunity and inflammation mediators is a key determinant of the histopathological changes and features of one disease or another. Anatomical sites may contribute to a disease pathogenesis, also in the case of LS [123]. With specific reference to VLS, with due distinctions, its physio-pathological process resembles that underlying morphea. An inflammatory driven fibrosis is considered the main pathogenetic event of morphea [124,125]. This could be a pathogenetic model shared by VLS. Consistent with this, the histopathological features of the two disorders present similar aspects. Moreover, genital LS has been found to be significantly more frequent in patients with morphea than in controls, with a frequency of 38% in patients with morphea [126]. A recent cross-sectional study found the frequent coexistence of extragenital LS and genital morphea [127]. These epidemiological data, together with the aforesaid clinical and histological similarities, seem to further support the strong link between the two clinical entities, which is probably determined by pathogenetic similarities.

## 3. Treatment Options

A definitive cure for VLS does not exist, as its exact etiopathogenesis remains unknown. The ideal treatment should aim at inducing relief of symptoms, reversing signs and preventing further anatomical changes, including tumorigenesis. Due to its progressive course and potential evolution towards cancer, all VLS cases should be treated, even when asymptomatic. The chronic nature of VLS is one of its main troublesome features, because recurrences are unavoidable when treatments are discontinued. Therefore, long-term maintenance of sign and symptom remission, achieved with an effective initial therapy, is an additional objective of treatment. 

Considering the recognized main pathophysiological events of VLS, the following two main potential therapeutic targets can be identified:

(1) auto-immunogenic mechanisms, and subsequent inflammation and oxidative stress.
(2) sclerotic tissue formation.

VLS treatment should act on one or both of these mechanisms.

Currently, there is no single option that can be recommended for the treatment of VLS. Several strategies are available and the choice depends on various factors. These include both disease-related aspects, mainly its length and severity, in terms of clinical features and symptoms, and patient variables such as age, expectations and quality of life impairment. Treatment options vary as well, depending on the phase of therapy, namely either acute or maintenance therapy.

The available treatment options are subdivided below on the basis of their main target, namely immune response and inflammation or collagen metabolism (Table A1 and Table A2).

### 3.1. Treatments Mainly Acting on Immune Dysreactivity and Inflammatory Response

#### 3.1.1. Topical Corticosteroids

High potency topical corticosteroids represent the first-line treatment in the active phase of VLS [1,128,129,130]. Topical corticosteroids’ effectiveness lies in their anti-inflammatory effect via interaction with the intracellular glucocorticoid receptor and the inducement of specific genes encoding signalling proteins with an immunosuppressive action. They exert an antipruritic effect, as well. Consistent with this, there is strong evidence that potent and ultra-potent corticosteroids have an excellent effect on both symptom control and reversal of histopathological and clinical features.

Topical clobetasol propionate 0.05% ointment or cream is the gold standard treatment for VLS, based on a long history of use [108,131,132,133] and wide clinical experience, as well as on several randomized controlled trials (RCT) [134,135,136,137,138,139,140]. Mometasone furoate 0.1% is another molecule whose effectiveness in VLS is supported by quite strong evidence [139,141,142,143]. Up to date, limited evidence on the effectiveness of alternative topical corticosteroids is available. In the initial treatment phase, 12 weeks is the duration of a corticosteroid treatment recommended for achieving a satisfying result with a favourable safety profile and tolerability. Topical corticosteroids can be used daily (once or twice, based on the molecule) for the entire 12-week duration. Tapering regimens have been suggested in order to avoid tachyphylaxis and reduce the risk of dose-dependent side-effects [129]. An RCT showed that in the initial VLS treatment, topical corticosteroids can be administered equally well in a continuous or tapering regimen [144]. In fact, both treatment regimens showed similar efficacy and tolerability. The ointment vehicle seems to optimize the therapeutic action of potent corticosteroids [145]. Local adverse effects potentially induced by the prolonged use of corticosteroids, such as atrophy, telangiectasia, or infections, usually do not occur with a 12-week therapy. Intralesional steroid injection is an alternative approach, to be reserved for cases resistant to topical treatment [146,147]. Setting up an effective long-term maintenance therapy, after an initial attack phase, is of paramount importance in the adequate therapeutic management of a chronic disease, such as VLS. With this specific goal, i.e., for preventing or, at least, delaying disease recurrences, topical corticosteroids can be administered

(1) on an “as needed” basis (“reactive” scheme) [129,143]
(2) on a continuative prolonged regimen [13,14,148,149,150],
(3) on a low-dose, intermittent regimen (“proactive” scheme) [151,152].

#### 3.1.2. Topical Calcineurin Inhibitors

Tacrolimus and pimecrolimus are calcineurin inhibitors used in topical preparations in the treatment of atopic dermatitis. By virtue of their immunosuppressive activity, topical calcineurin inhibitors (TCI) have been assessed in the treatment of many immune-mediated skin disorders, including VLS. TCI have been shown to be very effective and safe in treating VLS, in both adult and prepubertal patients [136,137,153,154,155,156,157,158,159,160,161,162,163,164,165]. Both tacrolimus and pimecrolimus, applied twice a day for 8 to 24 weeks, resulted highly effective in improving VLS signs and symptoms, with considerable complete response rates. A burning sensation, usually mild and transient, at the point of application during the first weeks of treatment, may occur. Patients should be informed of this so that they do not discontinue therapy. Since comparative trials have shown that ultra-potent corticosteroid is superior to TCI [136,137], tacrolimus and pimecrolimus are considered an effective alternative to strong topical corticosteroids in the treatment of active VLS. With reference to the concerns of potentiating squamous cell carcinoma occurrence with the use of TCI in patients with VLS, based on the available evidence, short-term application of TCI does not seem to increase the risk of squamous cell carcinoma in these subjects.

#### 3.1.3. Miscellaneous Topical Treatments 

Calcipotriol (CPT) is a synthetic derivative of calcitriol, the active form of vitamin D, which binds to the vitamin D receptor and exerts a gene regulatory effect. This leads to both attenuation of the abnormal keratinocyte proliferation and differentiation and inhibition of the inflammatory response induced by T cells, especially in psoriatic lesions [166]. CPT 0.005% ointment, applied once to twice a day for 16 weeks, induced a significant improvement, mainly in symptoms, in 8 women affected with VLS [167]. 

Oxatomide 5% gel is a molecule with antihistamine and anti-inflammatory properties. In a double-blind, cross-over, controlled trial that enrolled 22 VLS patients, oxatomide 5% gel, applied twice daily, was compared with placebo. It induced a significant improvement in itching compared with placebo but without relevant effects on clinical features [168]. 

Human fibroblast lysate cream (HFLC), also known as cutaneous lysate, is obtained from cultured human foetal fibroblasts. It has been shown to contain anti-inflammatory cytokines, such as interleukin 1 receptor antagonist (IL-1RA), IL-10, and IL-13. It also contains wound-healing growth factors, like EGF, FGFs and VEGF. In a small double blind, placebo-controlled trial including 30 VLS patients, HFLC applied for 12 weeks did not provide significant improvement compared to placebo [169].

#### 3.1.4. Systemic Treatments

Systemic agents exerting immunosuppressive or immune-modulating action, such as cyclosporine and methotrexate, are not supported by adequate evidence in the treatment of VLS. Thus, they may be considered an alternative therapeutic option exclusively in severe and refractory forms of VLS.

A single open-label trial which enrolled five VLS patients assessed efficacy and safety of oral cyclosporine, administered at 3 or 4 mg/kg/day for 12 weeks [170]. At the end of the third month, subjective symptoms regressed significantly and the clinical findings showed marked improvement. No severe side effects were observed. 

There are no studies that have specifically assessed methotrexate in the treatment of LS affecting the vulva. Considering a few trials carried out on extensive skin forms of lichen sclerosus, in some cases also involving the genitals, methotrexate, 10 to 15 mg/week for 6 months, showed improvement of clinical features [171,172]. In the study by Kreuter et al., methotrexate was administered with pulsed intravenous high-dose corticosteroids, for 3 consecutive days, monthly [172]. 

Promising perspectives, albeit supported by anecdotal experiences, derive from drugs capable of blocking the immune cascade by acting on novel targets, such as janus kinase (JAK) 1/2, which is inhibited by baricitinib [173].

### 3.2. Treatments Mainly Acting on Abnormal Fibroblast and Collagen Metabolism

#### 3.2.1. Topical Retinoids

Topical retinoids are therapeutic options for the treatment of VLS due to their normalizing action on both the keratinization process [174] and collagen metabolism [175]. Retinoids exert a mild anti-inflammatory effect as well [176]. Moreover, they have a role in the prevention of some skin cancers [177]. 

Up to date, two non-comparative studies have found that tretinoin 0.025% cream, applied either once a day, 5 days per week [178], or every other day [179] for prolonged periods, i.e., 6 to 12 months, induced improvement in subjective and clinical parameters and, when assessed, also in histological changes [178]. In another open, non-comparative study, 20 VLS patients were treated with 0.5% cis-retinoic acid in ointment [180]. Complete remission of clinical features was observed in 11 patients, usually after 1–2 months of daily retinoid application. Other single case reports can be found in the literature [181]. In the available literature, topical tretinoin produced an “irritant retinoid reaction”, mostly transient mild erythema with a burning sensation, in about one third of patients [178,179]. In an open label, nonrandomized, comparative study, tretinoin 0.05% cream, in short-contact therapy, was combined with mometasone furoate 0.1% ointment applied for 5 consecutive days a week, for 12 weeks. This treatment protocol was compared with the same corticosteroid, administered with the same regimen, simply combined with an emollient [182]. Surprisingly, no significant differences as regards the rate of response were found between the two study groups [182,183]. On the other hand, occurrence of local side effects, mostly burning, was higher in the patients treated with mometasone furoate plus tretinoin than among those treated with mometasone plus cold cream. This study shows that adding a topical retinoid, in short-contact therapy, to a potent corticosteroid does not enhance its effectiveness in the treatment of VLS. A less favourable safety profile of this combination, which may reduce patient adherence to the treatment, may explain the lack of therapeutic benefits of combining complementary topical molecules.

Therefore, based on the available literature, topical retinoids may represent a third-line choice, due to the lack of robust scientific evidence and their well-known local adverse effects. 

#### 3.2.2. Miscellaneous Topical Treatments

In the literature, avocado/soybean unsaponifiable (ASU), which is an extract prepared from avocado and soybean oil, has been shown to exert an anti-inflammatory effect by reducing several cytokines, prostaglandin E2, metalloproteinases and other pro-inflammatory mediators [184,185]. Moreover, when administered percutaneously on the dorsal skin of hairless rats for 15 days, it produced a modification of dermal connective tissue components [186]. In fact it induced an increase of soluble collagen and a decrease of insoluble collagen, as a result of the activation of connective tissue metabolism, mainly the inhibition of lysyl oxidase activity. The ASU action on the skin collagen metabolism may explain the beneficial effect on scleroderma observed by some authors [187,188,189]. An open-label, non-comparative study assessed efficacy and tolerability of a topical product containing avocado and soybean extracts and other lenitive and anti-oxidant principles administered for 24 weeks, in association with a dietary supplement containing avocado and soybean extracts vitamin E and para-aminobenzoic acid for the first 12 weeks, in the treatment of active mild-to-moderate VLS. Among the 23 women included, mean symptom and sign scores decreased significantly after treatment. The treatment was well tolerated [190]. Emollients and moisturizers do not have a direct action to improve the histopathological features of VLS, but rather to make the skin softer while preserving its integrity. They provide relief of symptoms and seem to delay flares [191,192]. This latter property makes them particularly suitable for long-term maintenance therapy.

#### 3.2.3. Physical Treatments

##### Phototherapy

The most convincing data on the potential benefits of phototherapy in VLS treatment concern UV-A1 phototherapy. UV-A1 phototherapy has been shown to be effective in a variety of sclerotic skin disorders, such as morphea, systemic sclerosis, eosinophilic fasciitis and necrobiosis lipoidica [193], which share some histological and clinical similarities with LS. UV-A1 enhances MMP activity, inhibits collagen synthesis and induces the fibroblasts’ release of cytokines which lead to an up-regulation of collagenase activity [193]. Moreover, it has strong melanogenic properties, which induce a re-pigmentation of LS pale surfaces [194]. 

A randomized trial compared clobetasol propionate 0.05% ointment, applied once daily for 12 weeks, with medium-dose UV-A1 (50 J/cm^2^) home-based phototherapy, performed four times weekly for 12 weeks [195]. At the end of treatment, UV-A1 phototherapy resulted in a significant clinical improvement, but was inferior to clobetasol propionate with respect to practicability, relief of itching, and improvement in quality of life. In a non-comparative study which included seven VLS patients, UV-A1 phototherapy was given three to five times/week for variable periods, with an acceptable clinical response in five patients [195]. Case series and anecdotal reports showed that 8-methoxypsoralen (8-MOP)-containing cream plus UVA (PUVA) as well as narrow band UVB induced some improvements in VLS-related symptoms and signs [196,197]. Some concerns remain about the use of phototherapy in VLS treatment, especially UVB and PUVA therapy, due to its well-known carcinogenic potential. 

##### Photodynamic Therapy

The pathophysiologic rationale of photodynamic therapy (PDT) is based on the conversion of ALA and methyl δ-ALA into the photosensitizing metabolic products called protoporphyrins. By activating protoporphyrins with the appropriate wavelength, PDT induces production of ROS, like superoxide, and singlet oxygen [198]. These reactions ultimately result in apoptosis of lymphocytes and keratinocytes, as well as in alteration of the expression of both cytokines and MMPs, which play a role in skin remodelling. Thus, even if the exact mechanism of PDT in VLS treatment remains uncertain, it seems to mainly act on dermal sclerosis. 

To date, a single open-label RTD compared four sessions of ALA-PDT, administered at 2-week intervals, with clobetasol propionate 0.05% ointment, administered once daily for 8 weeks, in 43 VLS patients [140]. At the end of treatment, both groups showed improvement in terms of clinical signs and symptoms. ALA-PDT showed a longer remission duration and a higher complete response rate than clobetasol propionate.

Further data are provided by some noncomparative studies including prospective cohorts and case series [199,200,201,202,203,204,205,206,207]. The majority of the patients included in these studies were treated with 5-ALA before the application of radiation (5-ALA PDT), whereas the remaining patients with methyl-aminolevulinate (methyl-PDT). Red light (630–635 nm) was the most commonly applied light, followed by green light (495–570 nm). 

Even considering the differences in both treatment schemes and outcomes adopted in the available studies, PDT was shown to induce symptom relief in most treated patients. Improvement in objective features was less constant and appreciable. Relevant changes in dermoscopic features have been documented as well [207]. 

No major adverse effects were reported during therapy and the post-treatment period, with the exception of mild to moderate pain or burning during irradiation. Based on a systematic review of the literature, a protocol for PDT in VLS has been recently proposed [208]. It consists in 5% 5-ALA as a photosensitizer applied for 3 h under occlusion before irradiation at the dose of 120 J/cm^2^ with red light (590–760 nm) and intensity of 204 mW/cm^2^.

##### Laser

Lasers, both ablative and non-ablative, are expected to be a suitable option in VLS treatment due to some collagen remodelling changes induced by these tools. In particular, lasers lead to neovascularization, neocollagenesis, elastogenesis, and restoration of the trabecular architecture of collagen, as well as reduction of epithelial degeneration and atrophy [209].

A single RCT assessed efficacy and tolerability of non-ablative neodymium: yttrium aluminium garnet (Nd:YAG) laser treatment of active VLS in comparison with a topical corticosteroid [210]. More in detail, the patients in the laser group received three Nd:YAG laser treatments every 14 days, whereas the control group received topical betamethasone for 4 weeks with tapering dosage. Those treated with laser applied betamethasone one week before the first laser treatment, and then for further 3 weeks with a decreasing dosage. Patients in the laser group had significantly greater improvement in VLS symptoms, better patient satisfaction, and greater reduction of sclerosis than those in the corticosteroid group, at 1- and 3-month follow-up visits. Both the very short course of corticosteroid treatment in the control group and the concomitant use of the same corticosteroid, albeit for a shorter period, in the laser group as well should be taken into consideration when interpreting these results. 

Ablative lasers, such as carbon dioxide lasers, have been used for recalcitrant and/or severe VLS in non-comparative, case series, studies [211,212,213,214,215,216]. CO2 laser treatment, very heterogeneous among the different studies for energy, treatment depth and regimens, led to an almost constant improvement in signs and symptoms. Post-operative pain was a common report. 

The potential benefits of micro-ablative fractional radiofrequency (MFR) on epithelial trophism and symptom improvement have been assessed in VLS patients [217]. After two to three sessions of MFR, most participants reported an improvement in symptoms, which persisted for about 11 months (range: 7–16 months) after the treatment. At histological examination, type III collagen concentration significantly increased and was associated with symptom improvement.

A recent systematic review supported the efficacy and tolerability of lasers, particularly fractionated ablative lasers, as an adjunct to topical potent corticosteroids for symptomatic relief in refractory VLS [218].

#### 3.2.4. Injective Treatments 

##### Adipose-Derived Stem Cells and Platelet-Rich Plasma 

Adipose-derived stem cells (ADSCs) are deemed capable of inhibiting fibrosis and regenerating damaged tissue by remodelling the extracellular matrix, especially in fibrotic disorders [219,220]. Moreover, they exert anti-inflammatory and immune-modulatory activity, due to the presence of pluripotent mesenchymal stem cells, endothelial progenitor cells, T cells, B cells, mast cells and adipose-resident macrophages. Platelet-rich plasma (PRP) contains platelets, which release growth factors supposed to reduce inflammation and to promote mesenchymal cell proliferation, tissue repair and angiogenesis [221,222]. In keeping with their action on the immune system and extracellular synthesis, two altered conditions in VLS, some observational studies and reports have assessed their efficacy and safety for the treatment of this condition [223,224,225,226,227,228]. Overall, these small series, non-comparative studies reported favourable short-term and patient-reported treatment outcomes with both ADSCs and PRP. In particular, ADSCs and PRP may represent promising therapeutic perspectives, especially for scarring, atrophy and the other sequelae of VLS which are poorly responsive to topical therapies. Methodological biases, the small number of patients included and the lack of standardized interventions, as well as of standardized outcome measures, limit the strength of these results [227].

An RCT assessed the efficacy and safety of autologous PRP for the treatment of VLS in 30 patients [229]. Enrolled patients were randomized to receive either placebo (saline injections) (10 subjects) or two separate treatments of PRP 6 weeks apart (20 subjects). No significant differences were found between the two study groups as regards the histological and clinical outcome parameters considered in the study. 

A recent study compared the effectiveness of local injection of purified adipose-derived stromal vascular fraction (AD-SVF) compared with purified SVF plus PRP in two randomized groups of LS patients [230]. Each patient received two surgical procedures, in general anaesthesia, distanced by a 4-month period. The study assessed clinical features, symptoms and quality of life before and after the treatment. The authors found that both AD-SVF and AD-SVF plus PRP are safe and efficacious regenerative methods for LS, especially in early disease stages. The addition of PRP did not improve treatment outcome of SVF graft.

##### Heterologous Type I Collagen

Effectiveness and safety of intradermal injections of heterologous type I collagen (HT1C) have been recently assessed in 3 VLS patients not responsive to ultra-potent corticosteroid [231]. HT1C stimulates both fibroblast proliferation and the production of extracellular matrix proteins [232]. Four injective treatments at 2-week intervals provided substantial relief of symptoms and complete resolution of clinical features in all of the treated subjects. VLS remission was maintained with further injections every 2 months. None of the patients complained of adverse events. The small number of cases and the lack of a comparison render these results only preliminary, even though they are promising.

#### 3.2.5. Systemic Treatments

In a multi-centre RCT, acitretin, a synthetic monoaromatic retinoid, administered orally at daily dosage of 20 to 30 mg/day for 16 weeks, was compared with placebo [233]. At treatment completion, significant improvement in itching, clinical features, such as atrophy, hyperkeratosis, erosions and lichenification, and the extent of VLS lesions were observed in the acitretin-treatment group as compared with the placebo group. A few noncomparative studies assessed the efficacy of etretinate, for many years now no longer on the market [234,235]. 

Potassium para-aminobenzoate (PABA) is an intermediate in the bacterial synthesis of folate (Vitamin Bx), which has been shown to have a favourable effect in several connective tissue disorders and skin fibroses when taken orally. A double-blind, placebo-controlled trial tested oral PABA (3 g capsules four times daily for 8 weeks) in 25 patients (22 women) suffering from genital and extra-genital LS [236]. After treatment, improvements recorded among the patients treated with oral PABA were not significantly different from those of the placebo group.

## 4. Concluding Remarks and Research Agenda

The ultimate objective for VLS patients undergoing specific treatments is to be no longer impaired by the disease in such aspects of their daily lives as distressing symptoms and sexual dysfunction. Achieving objective normality of skin colour and texture is a further key endpoint of treatment. Moreover, treatment should ideally protect against the progression to more severe forms of the disease and development of vulvar malignant neoplasm.

Considering both the available evidence and their action against the main pathophysiological mechanism underlying VLS, that is immune dysreactivity and inflammation, high potency topical corticosteroids represent the first-line treatment in the active phase of the disease. Topical calcineurin inhibitors, which inhibit the immune cascade as well, are an effective and safe alternative. The other available therapeutic options, including medical, physical and, to a lesser extent, systemic treatments, which mainly affect VLS tissue changes, should currently be considered third-line, at least until they are supported by stronger evidence. Further RCT, longer follow up, and better standardization of protocols and outcomes are required to shed light on the real potential of these treatments.

A maintenance treatment is necessary and should always be planned in order to stabilize the improvement obtained with the initial treatment and prevent flares [130]. 

However, the available treatment strategies have been shown to moderately satisfy women affected with VLS [237]. In agreement with this, recent studies highlighted the fact that complete healing of VLS occurs in a minority of patients treated with standard pharmacological treatments, despite the achievement of major improvements in symptoms and signs [238,239]. This means that, at treatment conclusion, a substantial rate of patients may still have residual disease. Thus, persistent negative effects on their well-being and quality of life may be supposed. 

This may be due to the fact that, despite the advances in the knowledge of the pathogenetic mechanisms of VLS, many aspects and mediators remain unclear. Further investigation is needed to better define the following issues:

(1) The exact sequence of events underlying VLS pathogenesis;
(2) The key mediators involved in VLS immune response and those which, more than others, trigger an abnormal fibroblast and collagen metabolism; in other words, the agents that convert inflammation into fibrosis;
(3) To what extent keratinocytes and fibroblasts actively participate in VLS pathogenesis and how they interact; and
(4) How a genetic background predisposes certain individuals to an abnormal release of pro-inflammatory and pro-fibrotic mediators, in response to still not fully understood triggers.

Advancement in this knowledge will presumably lead to making key mediators and events become therapeutic targets. This is the requirement for the development of extremely selective drugs, such as biologics, capable of modulating or suppressing specific pathogenetic sequences. These highly selective molecules have changed the natural course of many skin diseases that were not optimally managed until recently, such as psoriasis or atopic dermatitis. It is conceivable, and desirable, that the increasingly detailed definition of the mediators of the VLS pathogenetic process may allow us to have more effective drugs for patients suffering from this disabling disease. 

## Appendix A

**Table A1:** Treatments mainly acting on immune dysreactivity and inflammatory response.

Treatment	Posology	Notes
Topical treatments		
Topical Corticosteroids - Clobetasol Propionate 0.05% Ointment or Cream - Mometasone Furoate 0.1% Ointment or Cream	Once or twice a day for 12 weeks	- first line treatment in the active phase - anti-inflammatory and immunosuppressive activity - effectiveness on both symptoms and objective features - tachyphylaxis and dose-dependent side effects may be avoided by tapering regimens - ointment formulation seems to be more effective in comparison with cream - intralesional corticosteroid injection in recalcitrant forms - long-term maintenance treatment (reactive, continuative or proactive regimens)
Topical Calcineurin Inhibitors - Tacrolimus 0.1% Ointment - Pimecrolimus 1% Cream	Twice a day for 8 to 24 weeks	- second-line choice with lower effectiveness than ultra-potent corticosteroid - immunosuppressive activity - effectiveness on both symptoms and objective features - possible transient burning sensation during the first weeks of treatment
Calcipotriol 0.005% Ointment	Once to twice a day for 16 weeks	- inhibition of inflammatory response - attenuation of abnormal keratinocyte proliferation and differentiation - effectiveness on symptoms - alternative to standard treatment (weak evidence)
Oxatomide 5% gel	Twice a day for periods of 14-days	- antihistamine and anti-inflammatory properties - effectiveness on both symptoms and objective features - alternative to standard treatment (weak evidence)
Human Fibroblast Lysate Cream	Twice daily for 12 weeks	- presence of anti-inflammatory cytokines and wound-healing grow factors - no more effective than placebo
Systemic treatments		
Oral Cyclosporine	3–4 mg/kg/day for 12 weeks	- immunosuppressive effect - regression of symptoms and improvement of clinical features in resistant case - weak evidence
Oral or Subcutaneous Metothrexate	10 to 15 mg/week	- immunosuppressive effect - regression of symptoms and improvement of clinical features in resistant case - weak evidence
Baricitinib		- inhibition of JAK 1/2 - anecdotal reports

**Table A2:** Treatments mainly acting on abnormal fibroblast and collagen metabolism.

Treatment	Posology	Notes
Topical treatments		
Topical Retinoids - Tretinoin 0.025% or 0.05% cream - cis-retinoic acid 0.5% Ointment	Daily application for 5 days per week or every other day, for 6 to 12 months	- normalizing keratinization process and collagen metabolism - mild anti-inflammatory effect - effectiveness on both symptoms and objective features - frequent irritant reaction with mild erythema and burning sensation - third-line choice
Cream Containing Avocado and Soybean Extracts	Daily application for 16–24 weeks	- modification of dermal connective tissue components - anti-inflammatory effect - effectiveness on both symptoms and objective features in mild-to-moderate forms
Emollients and Moisturizers	Daily application for months	- no effect on clinical and histopatological changes - preservation of skin integrity - effectiveness on symptoms - long-term maintenance therapy
Physical treatments		
UVA1 Phototherapy	medium-dose UV-A1, 4 times weekly for 12 weeks	- inhibition of collagen synthesis and upregulation of collagenase activity - induction of repigmentation - effectiveness on both symptoms and objective features - alternative to topical potent and ultra-potent corticosteroids
Photodynamic Therapy	2-week intervals for 6–8 weeks and irradiation with red (630–635 nm) or green light (495–570 nm)	- induction of apoptosis of lymphocytes and keratinocytes - alteration of cytokines and metalloproteinases expression - promotion of skin remodeling - effectiveness on both symptoms and objective features - mild-to-moderate pain or burning during irradiation - alternative to topical potent and ultra-potent corticosteroids
Laser	heterogeneous treatment schemes of non-ablative (Nd:YAG) and ablative (CO2) lasers	- induction of collagen remodelling due to neovascularization, neocollagenogenesis, elastogenesis, restoration of the trabecular architecture - improvement of epithelial degeneration and atrophy - effectiveness on both symptoms and objective features - post-treatment pain - alternative or complementary approach to topical potent and ultra-potent corticosteroids
Injective treatments		
Adipose-Derived Stem Cells	heterogeneous treatment schemes and protocols	- inhibition of fibrosis - regeneration of damaged tissue - anti-inflammatory and immune-modulatory activity - effectiveness on scarring, atrophy and the other sequelae of VLS which are poorly responsive to topical therapies - alternative or complementary approach to topical potent and ultra-potent corticosteroids
Platelet-Rich Plasma	heterogeneous treatment schemes and protocols	- promotion of mesenchymal cell proliferation, tissue repair and angiogenesis - reduction of inflammation - effectiveness on scarring, atrophy and the other sequelae of VLS which are poorly responsive to topical therapies - alternative or complementary approach to topical potent and ultra-potent corticosteroids
Heterologous Type I Collagen	four injective treatments at 2-week intervals; then, every 2 months, as maintenance	- promotion of fibroblast proliferation and collagen synthesis - weak evidence
Systemic treatments		
Acitretin	20 to 30 mg/day for 16 weeks	- improvement in itching and clinical signs - alternative in recalcitrant forms - weak evidence
Potassium Para-Aminobenzoate (PABA)	3g/day for 8 weeks	- improvement of skin fibroses - no more effective than placebo
